# Forecasting the cumulative number of COVID-19 deaths in China: a Boltzmann function-based modeling study

**DOI:** 10.1017/ice.2020.101

**Published:** 2020-04-02

**Authors:** Yuanyuan Gao, Zuqin Zhang, Wei Yao, Qi Ying, Cheng Long, Xinmiao Fu

**Affiliations:** 1 Provincial University Key Laboratory of Cellular Stress Response and Metabolic Regulation, College of Life Sciences, Fujian Normal University, Fuzhou City, Fujian Province, China; 2 Department of Civil and Environmental Engineering, Texas A&M University, College Station, Texas; 3 Department of Orthopaedics, Sichuan University West China Hospital, Chengdu City, Sichuan Province, China

## Abstract

The COVID-19 outbreak is ongoing in China. Here, Boltzmann function-based analyses reveal the potential total numbers of COVID-19 deaths: 3,260 (95% confidence interval [CI], 3187–3394) in China; 110 (95% CI, 109–112) in Hubei Province; 3,174 (95% CI, 3095–3270) outside Hubei; 2,550 (95% CI, 2494–2621) in Wuhan City; and 617 (95% CI, 607–632) outside Wuhan.

An outbreak of 2019 novel coronavirus disease (COVID-19) caused by SARS-CoV-2 is ongoing in China and has spread worldwide.^1,2^ As of March 19, 2020, there had been 80,967 confirmed COVID-19 cases and 3,248 deaths in China. The epicenter of the outbreak, Wuhan city and related regions in Hubei Province, has reported 67,800 confirmed cases and 3,132 deaths. Although the number of new confirmed cases has substantially decreased since February 13, 2020, and the outbreak appears to be approaching the late phase in China, grave concerns about the severity of the outbreak have arisen, especially how many patients will die overall. Here, we have estimated the potential total number of COVID-19 deaths by applying Boltzmann function–based regression analysis, an approach that we recently developed for estimating the potential total numbers of confirmed cases for both the ongoing SARS-CoV-2 outbreak and the 2003 SARS epidemic.^3^

## Methods

We collected data for analysis of the officially released cumulative numbers of deaths in mainland China, other provinces (outside Hubei), Hubei Province, Wuhan City, and other cities in Hubei (from January 21 to February 29, 2020). The data were fitted with the Boltzmann function and the Richards function (see the Supplementary Information online).

## Results

**Fig. 1.**
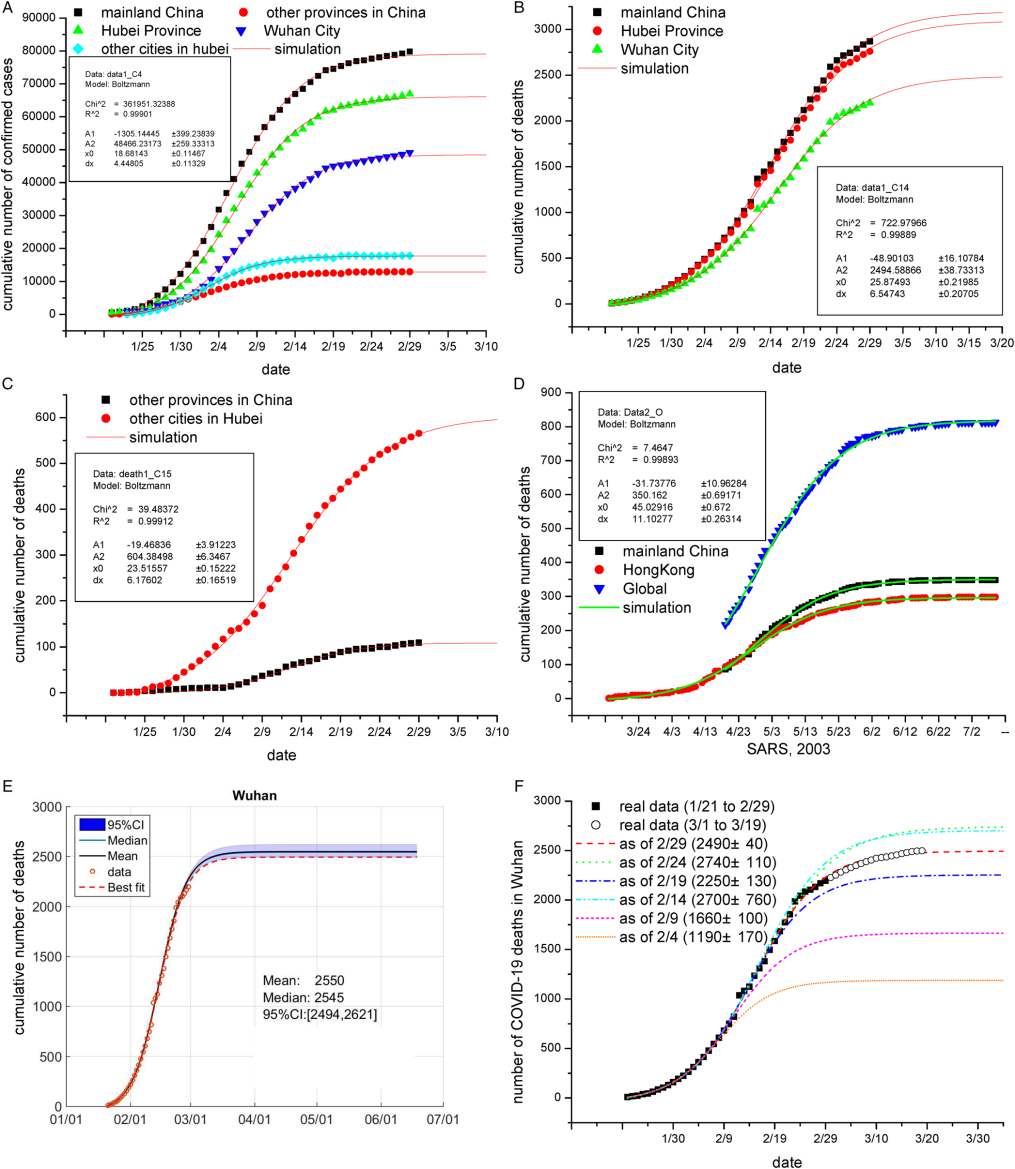
Fitting the cumulative number of COVID-19 deaths to Boltzmann function. (A–C) Boltzmann function-based regressions analysis results on the cumulative numbers of confirmed COVID-19 cases (panel A) and deaths (panels B and C) in the indicated geographic regions. Parameters of the established functions for Wuhan City (panels A and B) and for other cities in Hubei (panel C) are shown in insets. Note: The reported cumulative number of confirmed cases of Hubei Province and Wuhan City were readjusted for data fitting due to the suddenly added cases determined using clinical features (for details, refer to Supplementary Table 1 online). (D) Boltzmann function-based analysis results on the cumulative numbers of 2003 SARS deaths in the indicated regions. Parameters of the established function for mainland China are shown in insets. (E). Regression analysis results for COVID-19 deaths in Wuhan City using the Boltzmann functions assuming that the relative uncertainty of the data follows a single-sided normal distribution with a mean of 1.0 and a standard deviation of 2.5%. Original data are shown as circles; simulated results are presented as colored lines as indicated. Inserts show key statistics. Results for other regions are presented in Supplementary Fig. 1 (online). (F). Prediction of COVID deaths in Wuhan City by Boltzmann function-based analyses. The real data from January 21 to different closing dates were arbitrarily analyzed (colored lines), and the potential total numbers of deaths under these analyses are shown in insets. Real data (○) from March 1 to 19 agree well with the predicted data (dotted red lines) that were derived from the real data (▪) from January 21 to February 29.

We first verified that the cumulative numbers of confirmed cases with respect to each region were all well fitted to the Boltzmann function (ie, all *R* ^2^ close to 0.999) (Fig. 1A), consistent with our earlier report using the data from January 21 to February 14, 2020.^3^ Assuming that the number of deaths is proportional to the number of confirmed cases for the outbreak under specific circumstances, we speculated that the cumulative number of COVID-19 deaths would also obey the Boltzmann function. In support of this speculation, the cumulative numbers of COVID-19 deaths in the aforementioned regions were all well fitted to the Boltzmann function (*R* ^2^ all being close to 0.999) (Figs. 1B, 1C and Table 1). The potential total numbers of deaths were estimated to be 3,200±40 in China, 108±1 in Hubei, 3,100±40 outside Hubei, 2,500±40 in Wuhan City, and 604±6 outside Wuhan (Table 1). These results, in conjunction with our earlier observation that the cumulative numbers of confirmed cases of 2003 SARS in mainland China and worldwide were well fitted to the Boltzmann function, prompted us to analyze the cumulative numbers of 2003 SARS deaths in the same way. The cumulative numbers of 2003 SARS deaths in mainland China, Hong Kong, and worldwide were all well fitted to the Boltzmann function (Fig. 1D), strongly suggesting that the Boltzmann function is suitable to simulate the course of deaths associated with coronavirus-caused diseases.

**Table 1.** Summary of the Estimated Total Numbers of COVID-19 Deaths in China

Regions	Boltzmann^a^		Boltzmann, Mean (95% CI)^a^	Richards, Mean (95% CI)^a^	Real Data^b^
	Mean±SD	*R* ^2^			
Mainland China	3,200±40	0.999	3,260 (3,187–3,394)	3,342 (3,214–3,527)	3,248
Other provinces	108±1	0.996	110 (109–112)	111 (109–114)	116
Hubei Province	3,100±40	0.999	3,174 (3,095–3,270)	3,245 (3,100–3,423)	3,132
Wuhan City	2,490±40	0.998	2,550 (2,494–2,621)	2613 (2,498–2,767)	2,498
Other cities in Hubei	604±6	0.999	617 (607–632)	627 (603–654)	634

Note. SD, standard deviation; CI, confidence interval.

a Boltzmann function–based regression analysis results assuming the reported cumulative number of deaths (from January 21 to February 29) are precise and have uncertainty (SD, 2.5%), respectively (see the Methods section in the Supplementary Information online and Fig. 1E and Supplementary Fig. 1 online). The same uncertainty was set for Richards function–based regression analysis (see the Methods section and Supplementary Fig. 2 online).

b Officially reported cumulative numbers of COVID-19 deaths as of March 19, 2020.

One issue regarding our analyses is that some COVID-19 deaths might be misreported such that the reported numbers of deaths represent a lower limit. For instance, 134 new deaths were suddenly counted from >13,000 clinically diagnosed patients in Hubei Province on February 12, 2020 (as clearly indicated by a sudden increase in deaths) (Fig. 1B). Another uncertainty might result from those unidentified COVID-19 deaths that occurred in the early phase of the outbreak. We applied the Monte Carlo method (see the Methods section in the Supplementary Information online) to estimate such uncertainty assuming that the relative uncertainty of the reported numbers of deaths follows a single-sided normal distribution with a mean of 1.0 and a standard deviation of 2.5%. The potential total numbers of COVID-19 deaths were estimated to be 3,260 (95% confidence interval [CI], 3187–3394) in China; 110 (95% CI, 109–112) in Hubei Province; 3,174 (95% CI, 3095–3270) outside Hubei; 2,550 (95% CI, 2494–2621) in Wuhan City; and 617 (95% CI, 607–632) outside Wuhan (Fig. 1E and Supplementary Fig. 1 online). These numbers are slightly higher than those estimated without uncertainty (Table 1).

To verify our Boltzmann function–based estimates, we calculated the potential total numbers of deaths in the aforementioned regions by applying Richards function–based regression analyses, which were used to simulate the cumulative numbers of confirmed cases of 2003 SARS in different regions.^4,5^ The potential total numbers of COVID-19 deaths in mainland China, other provinces, Hubei Province, Wuhan City, and other cities were estimated to be 3,342 (95% CI, 3,214–3,527) in China; 111 (95% CI, 109–114) in Hubei Province; 3,245 (95% CI, 3,100–3,423) outside Hubei; 2,613 (95% CI, 2,498–2,767) in Wuhan City; and 627 (95% CI, 603–654) outside Wuhan (Supplementary Fig. 2 online). These simulated estimates are close to the numbers estimated by the Boltzmann function–based analyses (Table 1).

In further support of our estimates, we found that the established Boltzmann function was able to predict the death course in a short period such that the released cumulative deaths from March 1 to March 19 were close to the estimated numbers, for example, in Wuhan (Fig. 1F and Supplementary Table 1 online). When we analyzed the data from January 21 to different closing dates, we found that the courses of COVID-19 deaths could be largely be simulated based on the data through February 14 (Fig. 1F). These results indicate that the Boltzmann function-based analysis was able to predict the trend ahead by ~3 weeks.

## Discussion

Collectively, all sets of data from both COVID-19 deaths and the 2003 SARS deaths were well fitted to the Boltzmann function. We propose that the Boltzmann function is suitable for analyzing not only the cumulative number of confirmed COVID-19 cases, as we recently reported^3^ (Fig. 1A), but also numbers of deaths, as reported here. Modeling studies on the COVID-19 outbreak have been performed,^6^ and COVID-19 deaths have been estimated by other groups using different models. For example, using a data-driven analysis, Li et al^7^ recently predicted that total deaths in Hubei would be 2,250, much lower than the observed number, 2,761 (as of February 29). Using the susceptible–infected–recovered–dead model, Anastassopoulou et al^8^ predicted that total deaths might exceed 7,000 by February 29, but this number was much higher than the actual figure reported.

Because the crude fatality ratio in the epicenter of the outbreak is much higher than that in other provinces of mainland China,^1,2^ there is a great potential for government to optimize preparedness, therapeutic treatments, and medical resource supplies. In this way, hundreds of lives of COVID-19 patients, particularly severe and critically ill patients, might be saved.^2,9^ In addition, our estimates on the course of COVID-19 deaths (see Supplementary Table S2 online) may aid in providing mental health services to family members of those COVID-19 patients who pass away.^10^

## Supplementary material

For supplementary material accompanying this paper visit https://doi.org/10.1017/ice.2020.101. click here to view supplementary material
