# Supplementary material for: Forecasting the cumulative number of COVID-19 deaths in China: a Boltzmann function-based modeling study
Source: Infect Control Hosp Epidemiol. 2020 Apr 2:1–3. doi: 10.1017/ice.2020.101 (PMC7163192; doi:10.1017/ice.2020.101)
Supplement: Supplementary file 1 [file S0899823X20001014sup001.pdf]

Supporting information file

Forecasting the Cumulative Number of COVID-19 Deaths in China: a Boltzmann function-based modeling study

Zuqin Zhang, Cheng Long, Wei Yao, Yuanyuan Gao, Qi Ying and Xinmiao Fu

Methods

Sources of data

We collected the daily cumulative numbers of confirmed cases and deaths (from Jan 21, 2020 to Feb 29, 2020) of COVID-19 patients from official websites of the National Health Commission of China and Hubei Provincial Health Commissions. The cumulative numbers of confirmed cases and deaths of 2003 SARS in China and worldwide were obtained from the official website of WHO.

Data fitting with the Boltzmann function

Data were organized in Microsoft Excel and then incorporated into Microcal Origin software (note: Jan 21 2020 was set as day 1 and so on). The Boltzmann function was applied to data simulation for each set of data regarding different geographic regions (e.g.., China, Hubei Province and so on) and parameters of each function were obtained, with the potential total number of confirmed cases being directly given by parameter A_2. The Boltzmann function for simulation is expressed as follows:

$$C(x) = A_2 + \frac{A_1 - A_2}{1 + e^{(x - x0)/dx}} \quad (1)$$

where *C(x)* is the cumulative numbers of confirmed cases at day *x*; A_1, A_2, *x0,* and *dx* are constants. In particular, A_2 represents the estimated potential total number of confirmed cases. Details of derivation of the Boltzmann function for epidemic analysis are described in our recent report [1].

Furthermore, we assume that the number of deaths (designated as *D(x)*) is proportional to the number of confirmed cases (i.e., *C(x)*) under specific circumstances, although the former has a lag time behind the latter. Then, *D(x)* can also be expressed in the form of Boltzmann function.

Estimation of uncertainty in the non-linear regression

A Monte Carlo technique is applied to assess the uncertainty in the estimated total number of confirmed cases due to the uncertainty in the reported number cases. 1000 non-linear regressions were performed with the same time series data but each data point in the time series was perturbed by multiplying with a random scaling factor that represents the relative uncertainty. We assumed that the relative uncertainty follows a single-sided normal distribution with a mean of 1.0 and a standard deviation of 2.5%. This implies that all reported

cases are positive but there is a tendency to miss-reporting some positive cases so that the reported numbers represent a lower limit. The resulting mean and 95% confidence interval (CI) were presented.

Data fitting with the Richards function

The Richards function was applied to data simulation for each set of data regarding different geographic regions (e.g.., China, Hubei Province and so on) and parameters of each function were obtained, with the potential total number of confirmed cases being directly given by parameter b. The Richards function for simulation is expressed as follows according to earlier report [2, 3]:

$$I(x) = \frac{b}{(1 + e * exp^{-c(x-d)})^{1/e}} \quad (1)$$

where *I(x)* is the cumulative number of infected cases at day *x*; *b, c, d* and *e* are constants. In particular, *b* represents the estimated potential total number of infected cases. Furthermore, we assume that the number of deaths (designated as *D(x)*) is proportional to the number of infected cases (i.e., *I(x)*) under specific circumstances, although the former has a lag time behind the latter. Then, *D(x)* can also be expressed in the form of Richards function.

Results

Table S1 cumulative numbers of COVID-19 confirmed cases before and after adjustment and the numbers of deaths

date	mainland China					Hubei					Wuhan				
	a	*b*	*c*	*d*	*e*	*a*	*b*	*c*	*d*	*e*	*a*	*b*	*c*	*d*	*e*
1/21	440		440	608	9	375		375	543	9					9
1/22	571		571	770	17	444		444	643	17					17
1/23	830		830	1076	25	549		549	795	24	459		459	764	23
1/24	1287		1287	1614	41	729		729	1056	39	572		572	952	38
1/25	1975		1975	2447	56	1052		1052	1524	52	618		618	1029	45
1/26	2744		2744	3382	80	1423		1423	2061	76	698		698	1162	63
1/27	4515		4515	5732	106	2714		2714	3931	100	1590		1590	2647	85
1/28	5974		5974	7567	132	3554		3554	5147	125	1905		1905	3171	104
1/29	7711		7711	9767	170	4586		4586	6642	162	2261		2261	3764	129
1/30	9692		9692	12295	213	5806		5806	8409	204	2639		2639	4393	159
1/31	11791		11791	14997	259	7153		7153	10359	249	3215		3215	5352	192
2/1	14380		14380	18448	304	9074		9074	13142	294	4109		4109	6840	224
2/2	17205		17205	22215	361	11177		11177	16187	350	5142		5142	8560	265
2/3	20438		20438	26499	425	13522		13522	19583	414	6384		6384	10627	313
2/4	24324		24324	31800	490	16678		16678	24154	479	8351		8351	13902	362
2/5	28018		28018	36833	563	19665		19665	28480	549	10117		10117	16842	414
2/6	31161		31161	41073	636	22112		22112	32024	618	11618		11618	19340	478
2/7	34546		34546	45731	722	24953		24953	36138	699	13603		13603	22645	545
2/8	37198		37198	49346	811	27100		27100	39248	780	14982		14982	24940	608
2/9	40171		40171	53453	908	29631		29631	42913	871	16902		16902	28137	681
2/10	42638		42638	56860	1016	31728		31728	45950	974	18454		18454	30720	748
2/11	44750		44750	59707	1113	33366		33366	48323	1068	19558		19558	32558	820
2/12	59804	13332	46472	62105	1367	48206	13332	34874	50507	1310	32994	12364	20630	34343	1036
2/13	63851	15384	48467	64874	1445	51986	15384	36602	53009	1383	35991	14031	21960	36557	1079
2/14	66496	16522	49974	66956	1523	54406	16522	37884	54866	1457	37914	14953	22961	38223	1123
2/15	68500	17410	51090	68500	1665	56249	17410	56249	56249	1596	39462	15756	23705	39462	1233
2/16	70548			70548	1770	58182			58182	1696	41152			41152	1309
2/17	72436			72436	1868	59989			59989	1789	42752			42752	1381
2/18	74185			74185	2004	61682			61682	1921	44412			44412	1497
2/19	74576			74576	2118	62031			62031	2029	45027			45027	1585
2/20	75465			75465	2236	62662			63088	2144	45346			45346	1684
2/21	76288			76288	2345	63454			63454	2250	45660			45660	1774
2/22	76936			76936	2442	64084			64084	2346	46201			46201	1856
2/23	77150			77150	2592	64287			64287	2495	46607			46607	1987

2/24	77658			77658	2663	64786			64786	2563	47071			47071	2043
2/25	78064			78064	2715	65187			65187	2615	47441			47441	2085
2/26	78497			78497	2744	65596			65596	2641	47824			47824	2104
2/27	78824			78824	2788	65914			65914	2682	48137			48137	2132
2/28	79251			79251	2835	66337			66337	2727	48557			48557	2169
2/29	79824			79824	2870	66907			66907	2761	49122			49122	2195

a: reported cumulative number of confirmed cases.

b: cumulative number of confirmed cases determined by clinical features.

c: cumulative cases without those determined by clinical features.

d: adjusted cumulative number of confirmed cases for fitting are colored in red.

e: cumulative number of COVID-19 deaths for regression analysis.

Table S2 Prediction of the numbers of daily new COVID-19 deaths in Hubei Province and Wuhan City by the established Boltzmann functions [a].

**Date**	3/1	3/2	3/3	3/4	3/5	3/6	3/7	3/8	3/9	3/10	3/11	3/12	3/13	3/14	3/15
**Hubei**	40/42[b]	35/31	30/37	27/31	23/29	20/28	18/27	15/21	13/17	11/22	10/10	8/6	7/13	6/10	5/14
**Wuhan**	34/32	30/24	26/31	23/23	20/23	18/21	15/21	13/18	12/16	10/19	9/7	7/6	6/10	6/10	5/13
**Date**	3/16	3/17	3/18	3/19	3/20	3/21	3/22	3/23	3/24	3/25	3/26	3/27	3/28	3/29	3/30
**Hubei**	5/12	4/11	3/8	3/2	3	2	2	2	1	1	1	1	1	1	1
**Wuhan**	4/11	4/10	3/6	3/2	2	2	2	1	1	1	1	1	1	1	0

[a] Parameters of the established Boltzmann function for Hubei Province are as follows. A1: -70.604; A2: 3096.8; x0: 25.375; dx: 6.5043. Parameters of the function for Wuhan City are shown in the insert of **Fig. 1B**.

[b] The number of daily new deaths is colored in red for comparison with the predicted number.

Figure S1

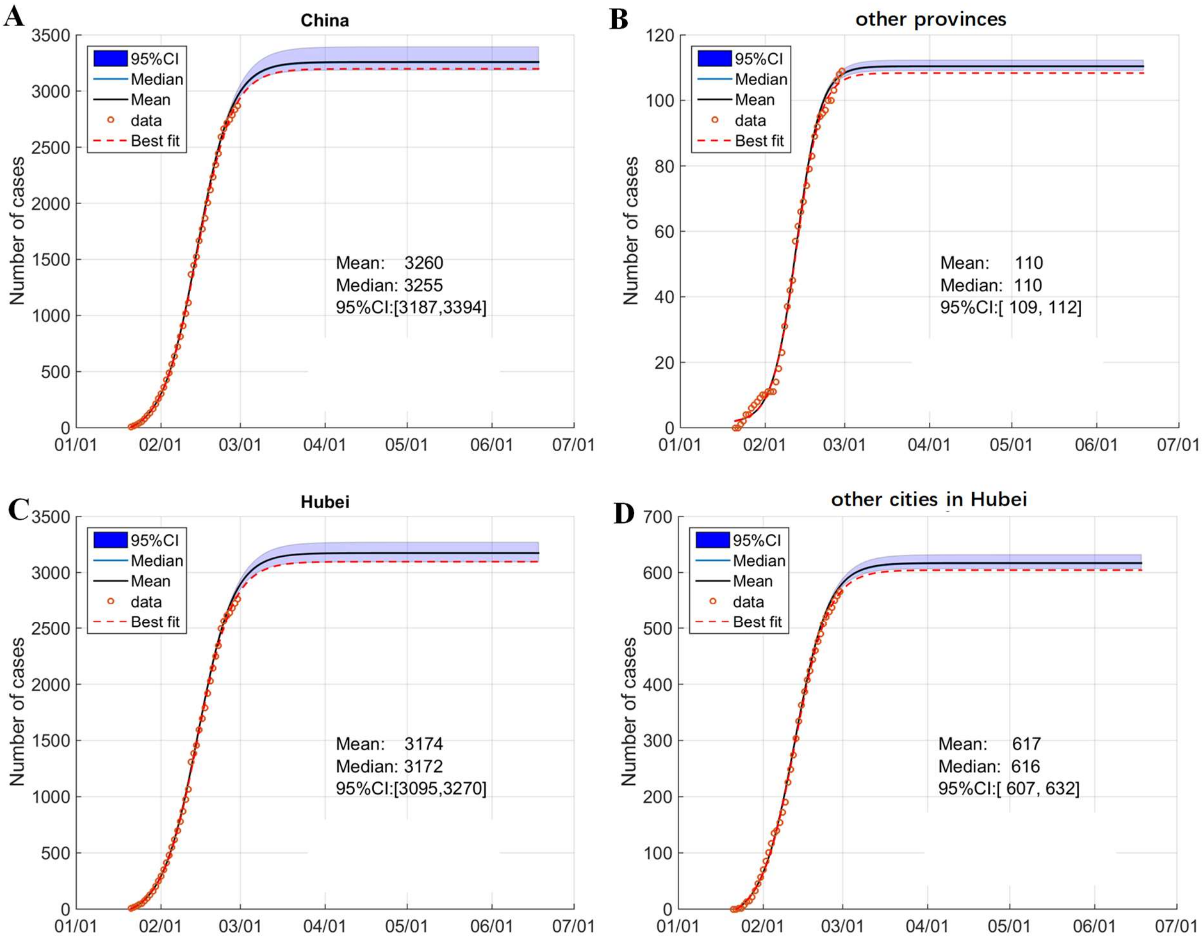

Figure S1. Analysis of the uncertainty of COVID-19 deaths for mainland China, other provinces, Hubei and other cities in Hubei using the Boltzmann function

Data of mainland China (panel A), other provinces (panel B), Hubei Province (panel C) and other cities in Hubei (panel D) were fitted to the Boltzmann function assuming that the relative uncertainty of the data followed a single-sided normal distribution with a mean of 1.0 and a standard deviation of 2.5%. The original data are shown as circles. The simulated results are presented as colored lines as indicated. The insets show key statistics. Results for Wuhan City is shown **Fig. 1E**.

Figure S2

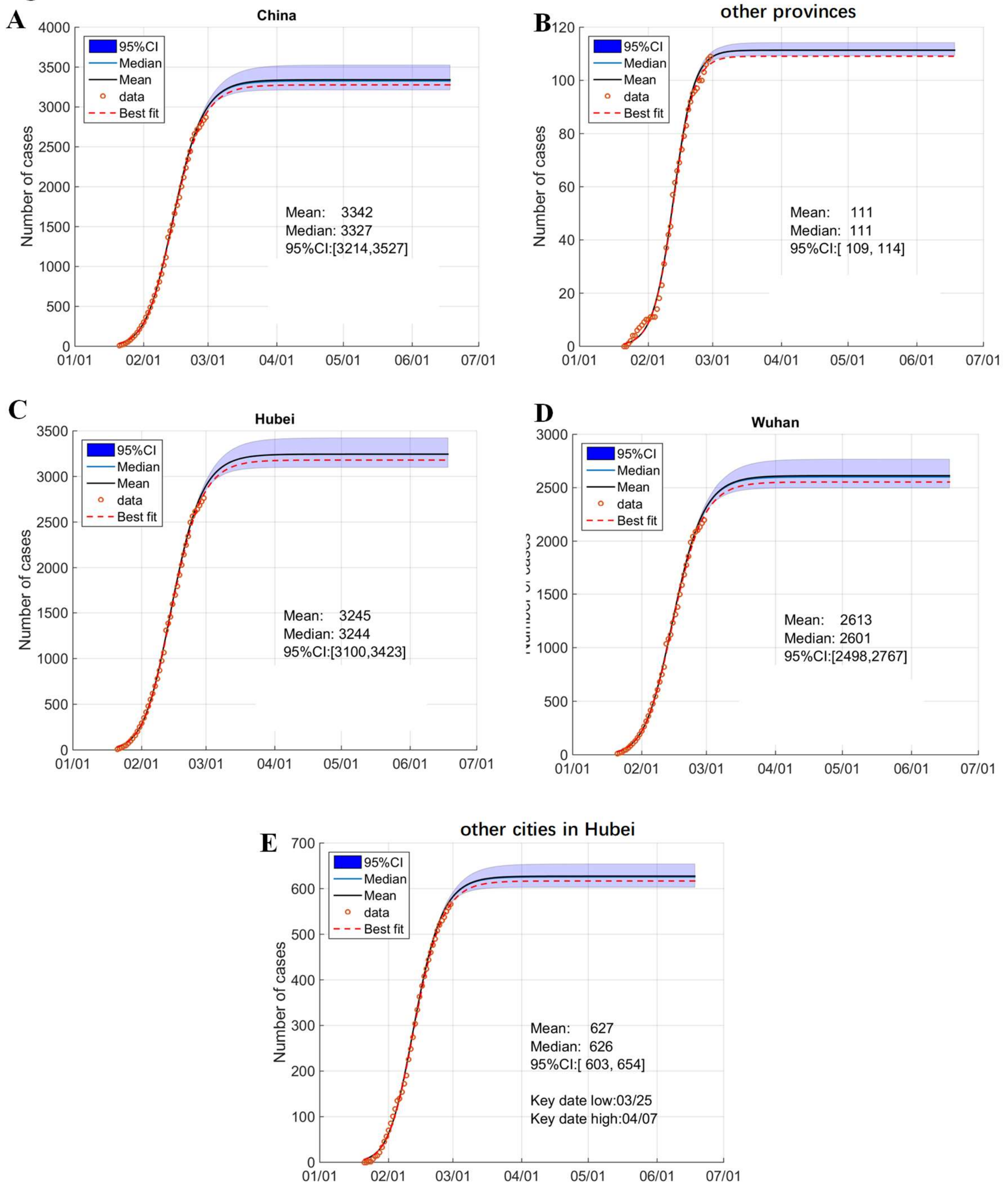

Figure S2. Analysis of the uncertainty of COVID-19 deaths using the Richards function

Data of mainland China (panel A), other provinces (panel B), Hubei Province (panel C), Wuhan City (panel D) and other cities in Hubei (panel E) were fitted to the Richards function assuming that the relative uncertainty of the data followed a single-sided normal distribution with a mean of 1.0 and a standard deviation of 2.5%. The original data are shown as circles. The simulated results are presented as colored lines as indicated. The insets show key statistics.

1. Fu, X., et al., Simulating and Forecasting the Cumulative Confirmed Cases of SARS-CoV-2 in

China by Boltzmann Function-based Regression Analyses. Journal of Infection, 2020; https://doi.org/10.1016/j.jinf.2020.02.019.
2. Wang, X.S., J. Wu, and Y. Yang, Richards modelrevisited:Validationbyandapplicationto infection dynamics. Journal of Theoretical Biology, 2012. 313 12–19.
3. Hsieh, Y.H., Richards Model: A Simple Procedure for Real-time Prediction of Outbreak Severity. Modeling and Dynamics of Infectious Diseases, 2009; p. 216-236 https://doi.org/10.1142/9789814261265_0009.
